# Supplementary material for: Effects of Biomaterials Derived from Germinated Hemp Seeds on Stressed Hair Stem Cells and Immune Cells
Source: Int J Mol Sci. 2024 Jul 17;25(14):7823. doi: 10.3390/ijms25147823 (PMC11276847; doi:10.3390/ijms25147823)
Supplement: Supplementary file 1 [file ijms-25-07823-s001.zip › supplementary data_S1. analytic results.pdf]

Supplementary data

: Alteration of compositions in germinated hemp seeds

Evaluation of CBDVA from cheongsam seeds and germinated seed, hemp in Andong city, Korea

- LC-MS/MS
Instrument: Agilent Technologies 6410 Triple Quad (LC-MS/MS)
Column: Unisol C18 (3.0 mm × 150 mm, 3.0 ㎛)
Solvent: A; 0.1% Formic acid in Water, B; 0.1% Formic acid in Acetonitrile
Flow rate: 0.4 mL/min
Injection vol.: 5 mL
Gradient :

	time(min.)	B%
**1**	0	70
**2**	12	100
**3**	15	100

- MS condition

Ionization Mode	-ESI, scan mode
**Gas temp.**	350 ℃
**Capillary volt.**	4000 V
**Nebulizer**	40 psig
**Fragmentor**	135 V

- CBDVA : CAS Number <u>31932-13-5</u>

Results

Standard (CBDVA)

HSE

GHSE

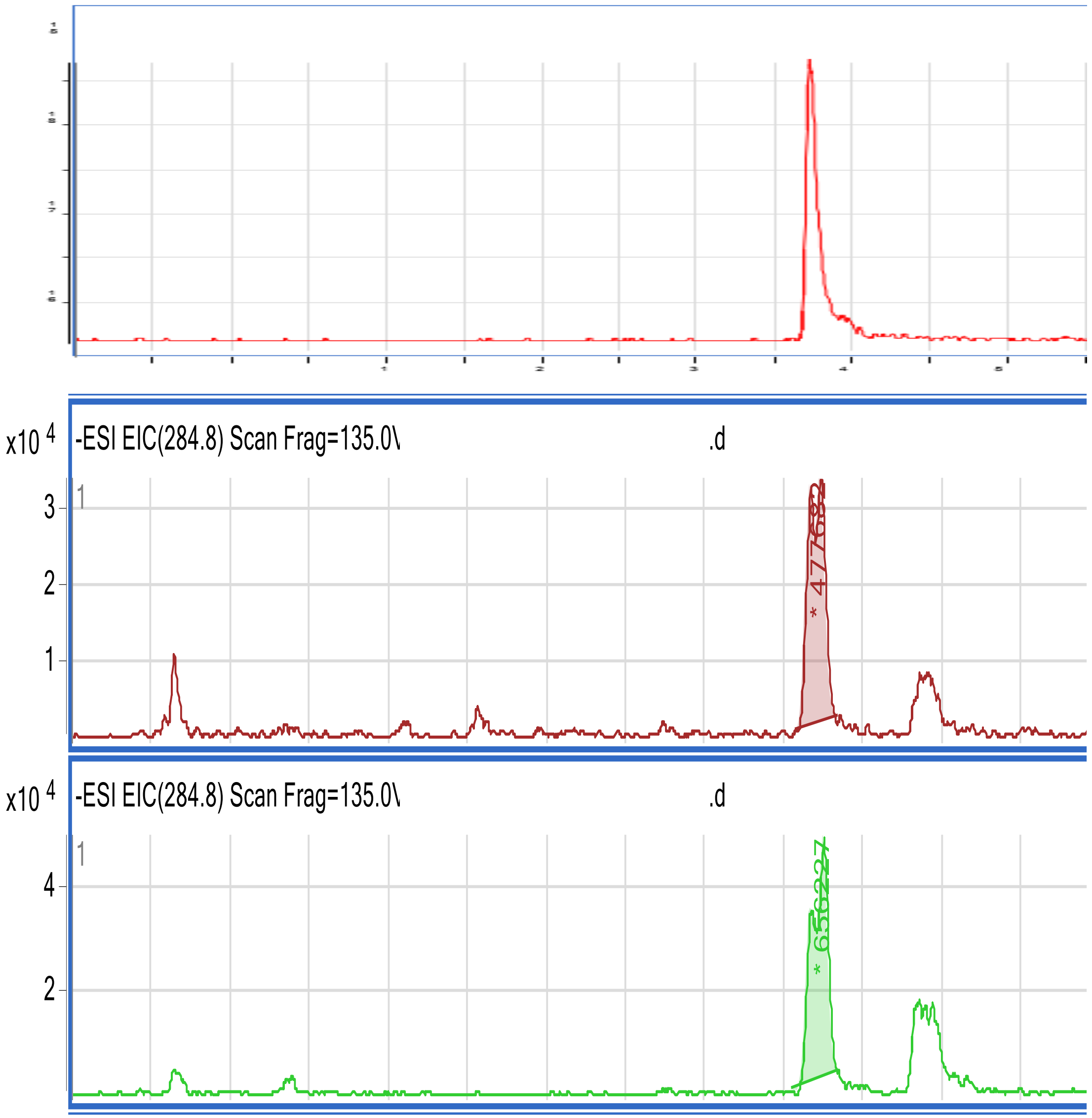

Calibration curve

Compounds	Regression equation	Linear range	Correlation coefficient
		(mg/mL)	(R^2)
CBDVA	y = 622,021.0959x - 5,764.0411	0.5-5	1.0000

Results

Sample		
	Area	Sample 1mg
HSE	477,682	0.016 mg
GHSE	656,227	0.021 mg

Saponin

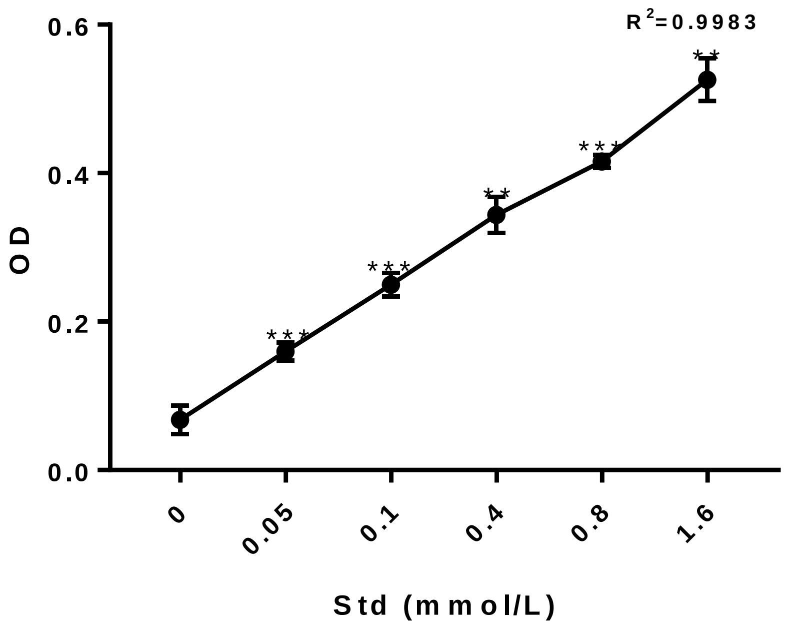

(** p < 0.01; *** p < 0.001)

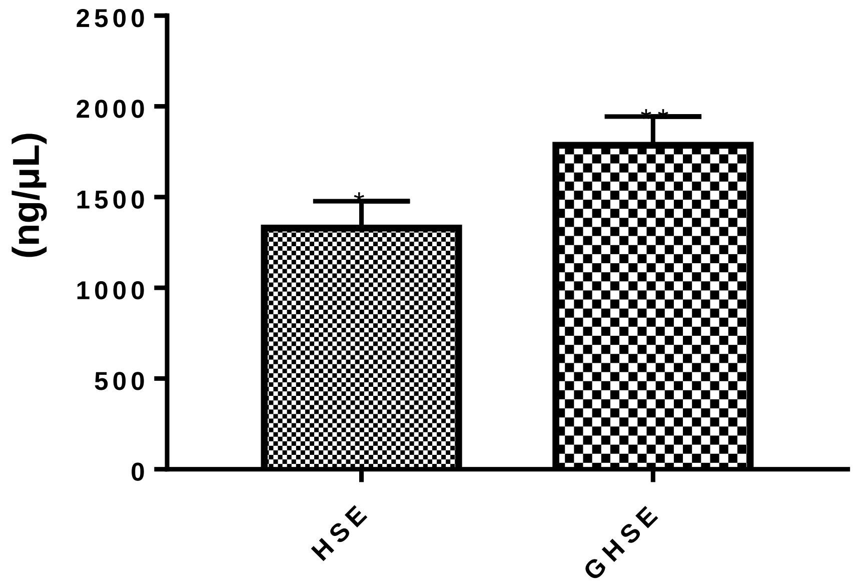

OD; optical density

HSE : hemp seed extract (50% ethanol)
GHSE : Germinated hemp seed extract (50%ethanol)

Ferulic acid

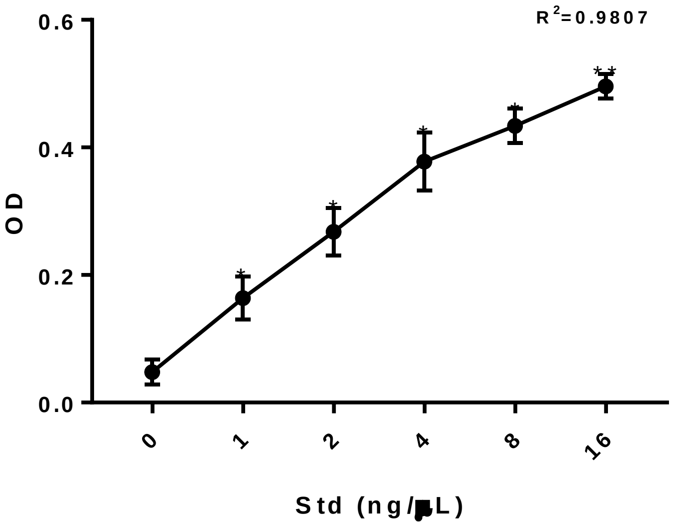

(* p < 0.05; ** p < 0.01)

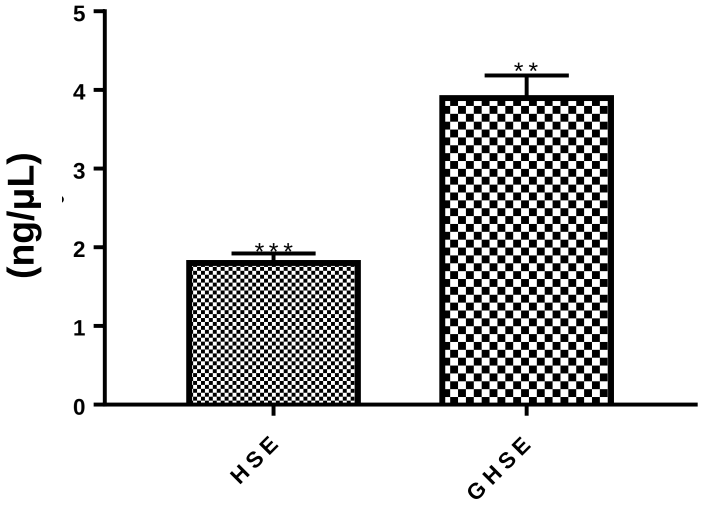

OD; optical density

HSE : hemp seed extract (50% ethanol)
GHSE : Germinated hemp seed extract (50%ethanol)
