# Supplementary figures and images for: Effects of Biomaterials Derived from Germinated Hemp Seeds on Stressed Hair Stem Cells and Immune Cells
Source: Int J Mol Sci. 2024 Jul 17;25(14):7823. doi: 10.3390/ijms25147823 (PMC11276847; doi:10.3390/ijms25147823)

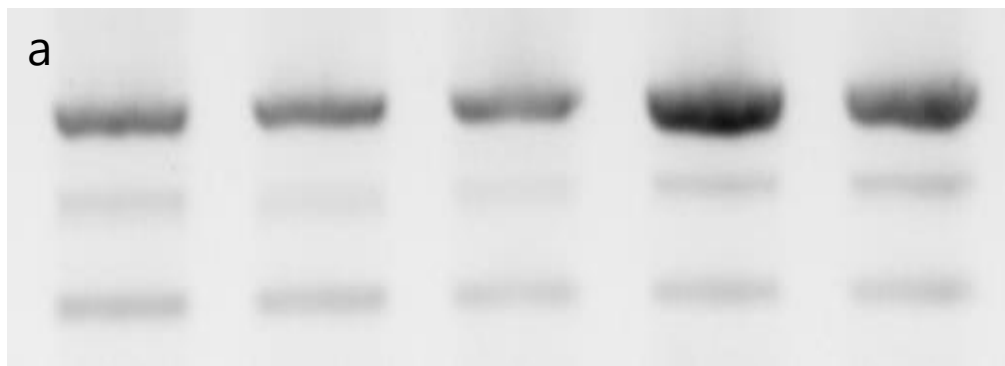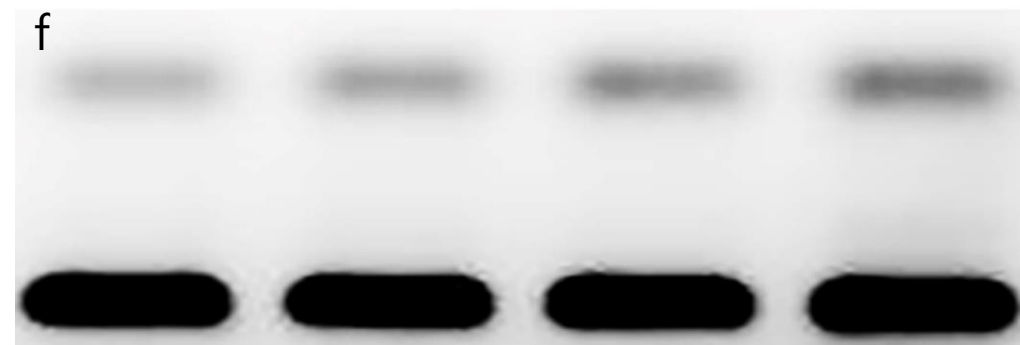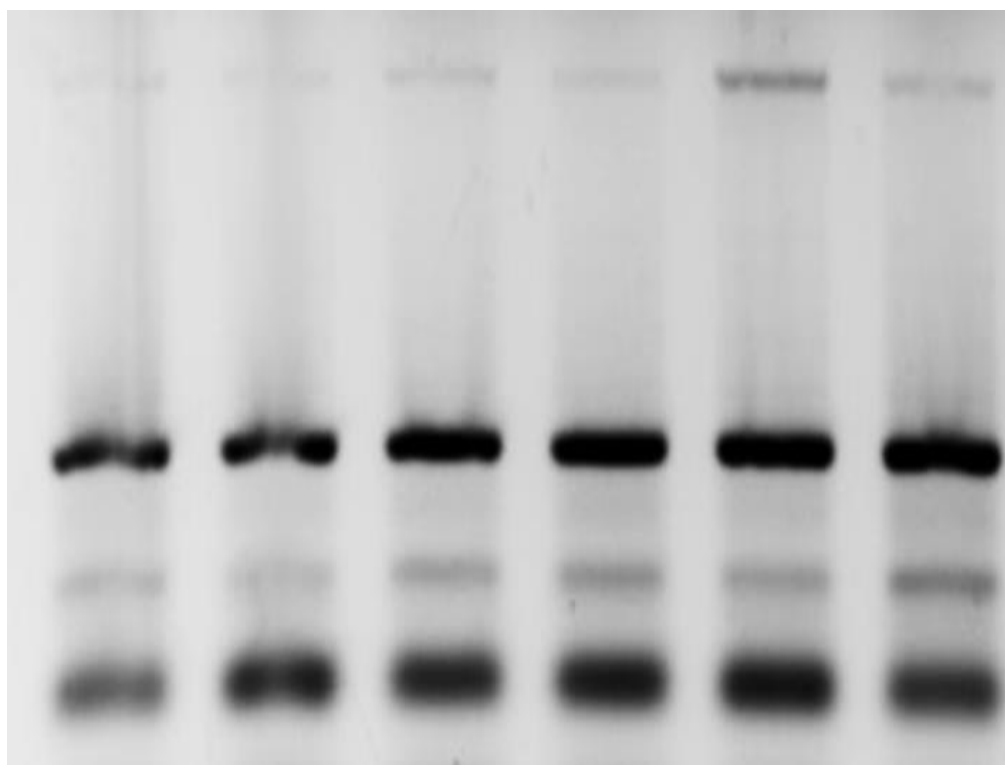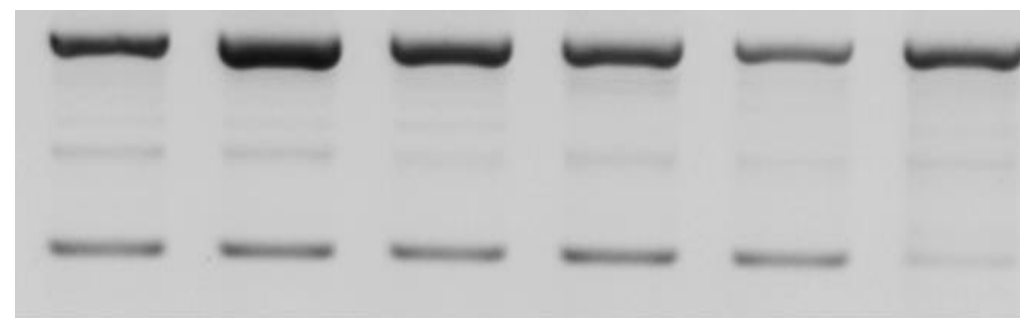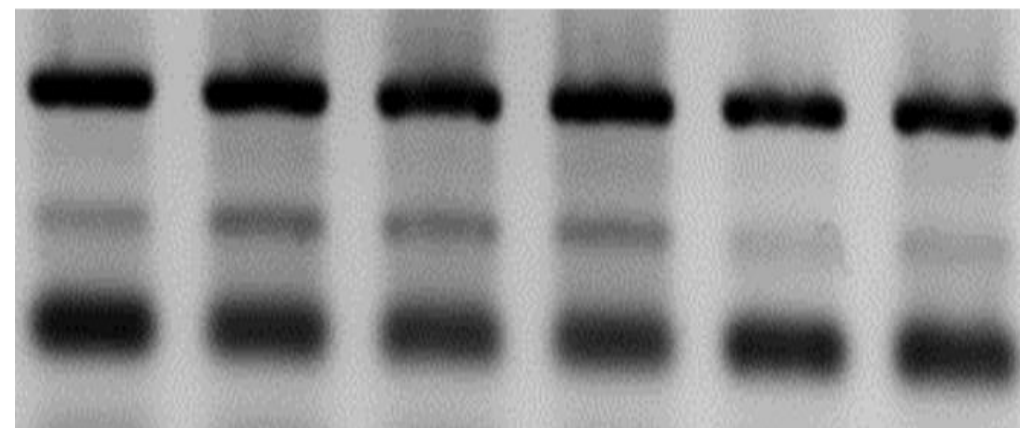

Full gels –fig1

Full gels –fig2

Full gels –fig3

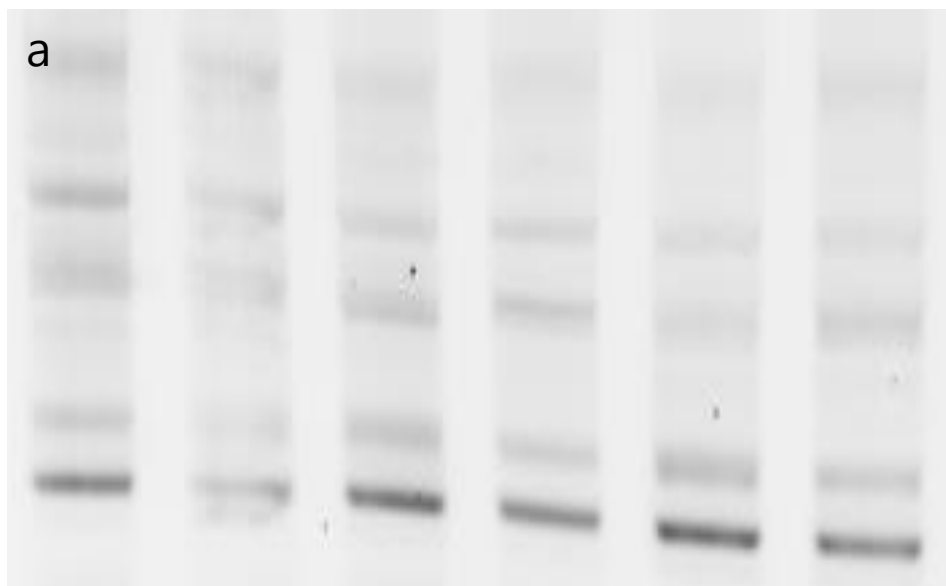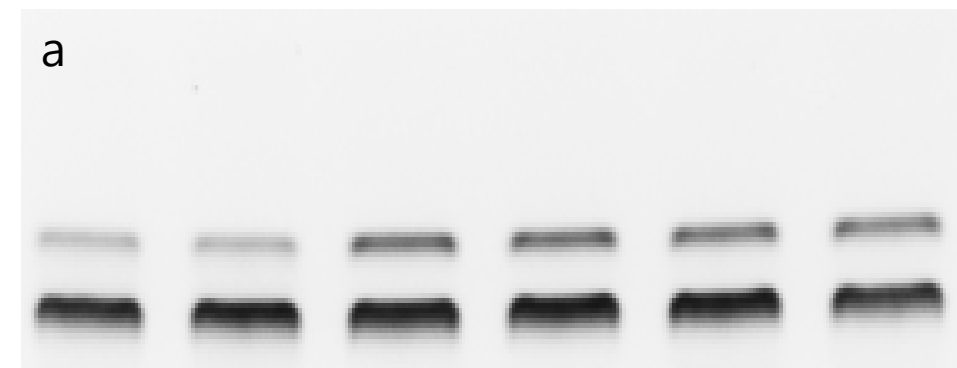

Full gels –fig7

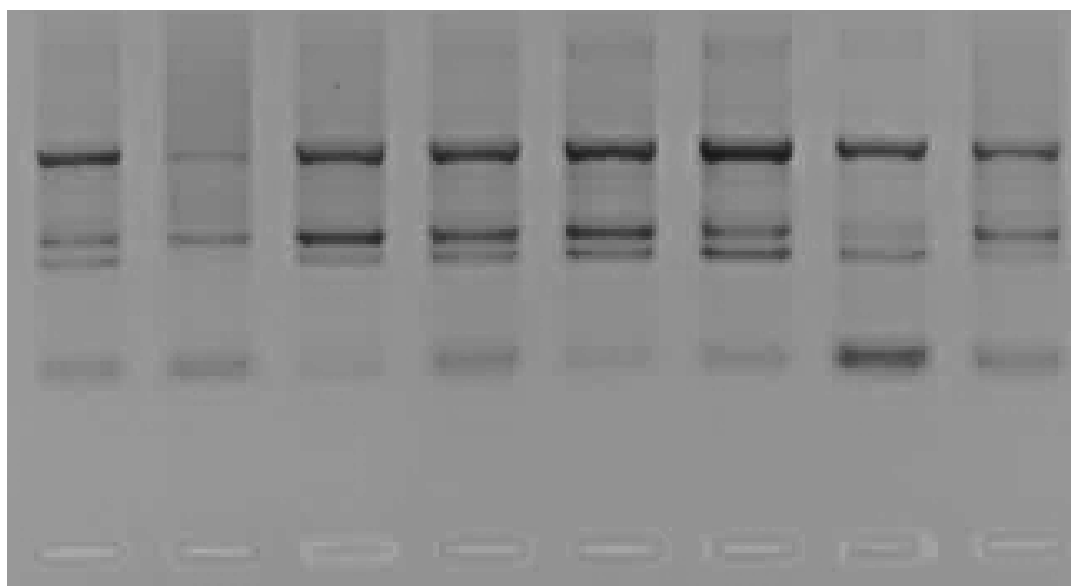

Supplement: Supplementary file 1 [file ijms-25-07823-s001.zip › full gels.pdf]
